# Correlation Between Neutrophil Count and Prognosis in STEMI Patients with Chronic Renal Dysfunction: A Retrospective Cohort Study

**DOI:** 10.1515/biol-2019-0075

**Published:** 2019-12-31

**Authors:** Yuhan Cao, Cong Fu, Xin Wang, Chaojun Yu

**Affiliations:** 1 Department of Nephrology, Yi Ji Shan Hospital Affiliated to Wannan Medical College, 92 West Zheshan Road, Wuhu 241001, Anhui, China; 2 Department of Nephrology, Zhongda Hospital Affiliated to Southeast University, Nanjing, China; 3 Departments of Cardiology, Yijishan Hospital Affiliated to Wannan Medical College, Wuhu, China; 4 Department of Cardiology, Zhongda Hospital Affiliated to Southeast University, Nanjing, China; 5 Department of Cardiology, Jiang Yin Peoples’ hospital, Jiang Yin, China

**Keywords:** neutrophil count, ST-elevation myocardial infarction, chronic renal dysfunction, clinical outcome

## Abstract

Neutrophil is a key element in inflammation and stress disease, which are associated with poor clinical outcomes in various cardiac diseases. However, the clinical availability of neutrophil in patients with ST-elevation myocardial infarction (STEMI) and chronic renal dysfunction has not been known. Accordingly, we designed this retrospective cohort study to evaluate the differences of major adverse cardiovascular events incidence between renal dysfunctional STEMI patients with normal and high neutrophil levels. The primary end point was all-cause mortality. We analyzed 377 consecutive STEMI patients with chronic renal dysfunction. The results showed that during 12-48 months follow-up, death from any-cause occurred in 1.4% patients (4 of 290) in normal-level neutrophil group, as compared with 3.4% in high-level neutrophil group (3 of 87) (hazard ratio, 2.174 95% confidence interval, 1.024-10.248; P = 0.025). Kaplan-Meier survival analysis showed that there were significant differences between the two groups with respect to the risk of death (P=0.018), and heart failure (P=0.037).

## 1 Introduction

Inflammation plays a key role in myocardial infarction (MI).. It has been demonstrated that inflammatory biomarkers such as C-reactive protein (CRP) are significantly associated with cardiovascular disease [1, 2]. In the pathological process of coronary artery disease, inflammatory factors can promote the atherosclerotic plaque rupture and thrombosis [3, 4]. Among the inflammatory factors, neutrophil count is widely known as classic inflammatory biomarkers to predict clinical outcomes of disease [5]. The previous study indicated that the neutrophil to lymphocyte ratio (NLR) has been evaluated as a prognostic biomarker for various cardiovascular diseases [6]. Additional, combined CRP and NLR can better indicate the prognosis of coronary artery disease [7]. Chronic renal dysfunction is common in myocardial infarction. The hypercoagulable state in chronic renal dysfunction usually leads to poor outcomes [8]. However, it remains unknown that if neutrophil can indicate the prognosis of myocardial infarction with chronic renal dysfunction. Consequently, it is important to identify if neutrophil is predictive to STEMI with chronic renal dysfunction. On account of these reasons, this retrospective cohort study was designed to identify if neutrophil count can indicate the prognosis of STEMI in chronic kidney disease. We examined 377 STEMI patients with renal dysfunction who have normal or high neutrophil. Clinical characters and follow-up data were collected. The incidence of Major Adverse Cardiovascular Events (MACEs) was evaluated to determine the predictive value of prognosis.

## 2 Methods

### 2.1 Study design

The study was a single center, retrospectively cohort study. Trial administration, data management, and statistical analyses were performed at the Department of Cardiology, Zhongda Hospital Affiliated to Southeast University. All the samples from patients enrolled in this study that diagnosed as STEMI with renal dysfunction was analyzed except for the patients who were in accordance with exclusion criterion.

**Informed consent**: Informed consent has been obtained from all individuals included in this study.

**Ethical approval**: The research related to human use has been complied with all the relevant national regulations, institutional policies and in accordance the tenets of the Helsinki Declaration, and has been approved by the Ethics Committee of Zhongda Hospital.

### 2.2 Patient population

A total of 377 patients with STEMI and chronic renal dysfunction were diagnosed by coronary angiography (CAG) in the Department of Cardiology, Zhongda Hospital from December, 2009 to December, 2011 were enrolled. Glomerulonephritis was diagnosed by biochemical data and/or renal biopsy. Estimate glomerular filtration rate (eGFR) was calculated by MDRD formula. Chronic renal dysfunction was defined as the eGFR <90 mL/min/1.73m^2^ except for some acute factors leading to acute renal injury The patients were separated into two groups according to the neutrophil counts. The definition of myocardial infarction was described previously [9]. Patients who have cancer, stroke, old myocardial infarction, received stents implantation before, severe liver dysfunction, chronic heart failure, autoimmune diseases, infection diseases, receiving hormone and/or immunosuppressant therapy and hemodialysis, died before discharge from hospital were excluded. Patients lacking of clinical documents were also excluded. Patients received drug therapy according to the situation of disease based on the guideline. All patients were asked to confirm their agreement to accept the 12-48 months follow-up by providing written informed consent.

### 2.3 Therapy procedures

All the patients that participated in the study underwent coronary angiography and 223 patients underwent percutaneous coronary intervention (PCI). Usage of platelet inhibitors or anticoagulants and other symptomatic treatment were decided by physician according to guideline and clinical condition. Clopidogrel and aspirin were administered as 600 mg and 300 mg once arrived in hospital and then immediately transferred to catheter room to receive CAG and/or PCI treatment. Clopidogrel and aspirin were administered 75mg and 100mg per day after PCI, respectively. A beta receptor blocker, statin and LMWH were administered according to the patients’ status and guidelines. Angiotensin converting enzyme inhibitors/angiotensin receptor blocker (ACEI/ARB) was administered in patients who had a serum creatinine level less than 256umol/L. Neutrophil counts was measured by clinical laboratory of Zhongda Hospital. Normal neutrophil group was defined as proportion of neutrophil (N%) ≤75%. High neutrophil group was defined as N%>75%.

### 2.4 End points

Major adverse cardiovascular events were recorded (MACEs). Death from any cause was defined as the primary end points which defined as death of cardiac causes or any death without another known cause. Coronary revascularization was defined as angioplasty or stenting or coronary artery bypass grafting. Heart failure was defined as BNP measured in Zhongda Hospital at least above the 5* 99^th^ percentile upper reference limit. MACEs were verified by hospital medical records and telephone. No missing data was generated during follow-up.

### 2.5 Statistical analysis

The data were analyzed using the statistical software package of SPSS (SPSS Inc., Chicago, IL, USA, Version 17.0). Numerical variables were expressed as mean±standard deviation and categorical variables as percentages. Continuous variables between groups were compared by unpaired Student’s t test. Categorical variables were compared by Chi-square test. Kaplanmeier survival analysis was performed. Hazard ratio (HR) and 95% confidence intervals (CI) were calculated by Cox proportional hazard model. Two-tailed P values <0.05 were considered significant.

## 3 Results

### 3.1 Study population

**Figure 1:**
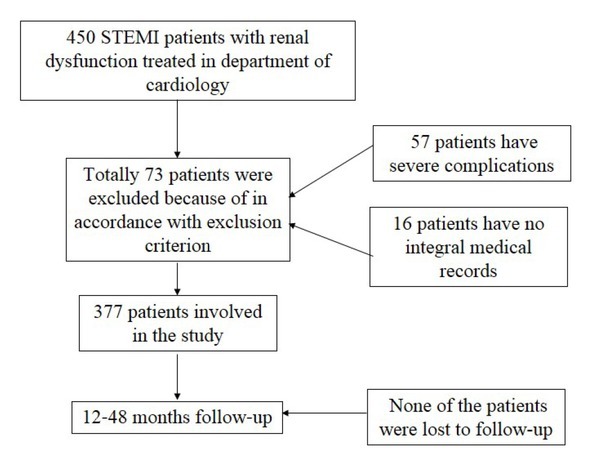
The flow diagram of this study.

**Figure 2:**
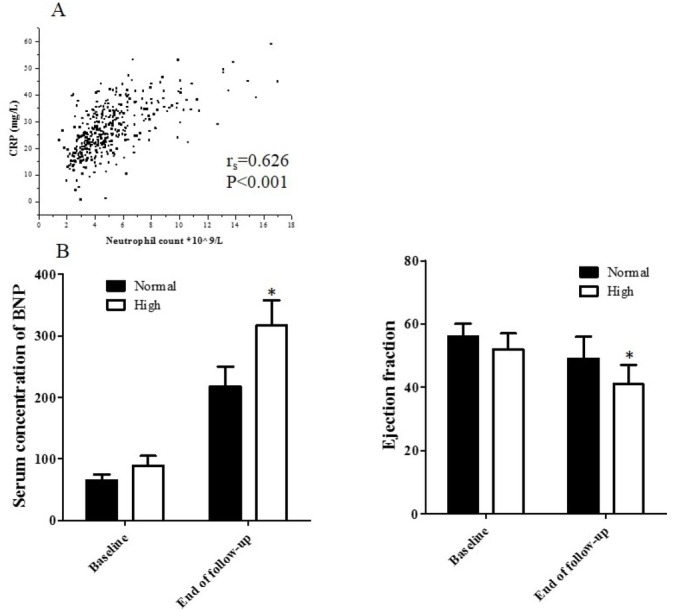
A: The correlation between CRP and neutrophil in all patients. B: The level of BNP and ejection fraction at baseline and end of follow-up in normal neutrophil group and high neutrophil group (*P<0.05 vs baseline).

Among 450 patients treated in the Department of Cardiology, 57 patients were excluded because of exclusion criterion. In addition, 16 patients were excluded because of incomplete medical records. Among all the 377 patients enrolled in the study, none of them have progressed to uremia. There were 290 patients involved in normal neutrophil group and 87 patients in high neutrophil group, respectively (**Figure 1**). None of these patients progressed to end stage renal disease or received maintenance hemodialysis. The baseline clinical characters and biochemical data, lesion coronary artery, complications and therapy were listed in Table 1. The correlation between C-reactive protein (CRP) concentration and neutrophil count was showed in figure 2 (r_s_=0.626, P<0.001). None of the patients were lost in follow-up with respect to the end point.

**Table 1:** Baseline characteristics of myocardial infarction patients with chronic renal dysfunction.

	Patients (n=377)		P
	Normal (n=290)	High (n=87)	
Sex, M/F	210/80	70/17	0.132
Age, y	66.5±10.5	69.2±10.9	0.044
WBC	7.9±3.1	7.9±3.4	<0.001
RBC	4.6±2.4	4.5±0.6	0.630
HB	135±18	134±17	0.649
Neutrophil	4.3±1.4	7.9±3.3	<0.001
Neutrophil ratio(%)	65.0±6.3	81.9±4.6	<0.001
CRP, (mg/L)	1.5 (0.7-47.3)	2.1(1.2-59.0)	0.067
Gensini score	75±12	76±23	0.384
BNP (pg/mL)	65±10	89±16	0.053
Ejection fraction (%)	56±4	52±5	0.159
Killip classification,n(%)			
Class I	120	32	0.239
Class II	78	19	
Class III	51	29	
Class IV	41	7	
cTnI	4.9±1.7	14.0±3.8	0.132
TC	4.3±1.0	4.3±1.0	0.567
TG	1.4±0.9	1.3±0.7	0.148
LDL	2.7±0.8	2.7±0.9	0.866
HDL	1.1±0.3	1.1±0.3	0.611
Scr, (umol/L)	156±18	164±21	0.321
BUN, (mmol/L)	19±7	21±9	0.248
eGFR	60±13	59±22	0.116
Smoking, n(%)	93(32.1)	17(19.5)	0.024
HP, n(%)	219(75.5)	65(74.7)	0.879
DM, n(%)	76(26.2)	26(29.9)	0.498
LM, n(%)	16(5.5)	9(10.3)	0.112
LAD, n(%)	172(59.3)	58(66.7)	0.217
LCX, n(%)	111(38.3)	39(44.8)	0.274
RCA, n(%)	109(37.6)	42(48.3)	0.074
PCI, n(%)	164(56.6)	59(67.8)	0.061
Aspirin, n(%)	275(94.8)	83(95.4)	0.830
Betaloc, n(%)	212(73.1)	65(74.7)	0.766
ACEI/ARB, n(%)	150(51.7)	48(55.2)	0.572
Statin, n(%)	254(87.6)	79(90.8)	0.412
LMWH, n(%)	144(49.7)	52(59.8)	0.098
Clopidogrel,n( %)	173(59.7)	65(74.7)	0.011

Normal: normal neutrophil count group. High: high neutrophil count group. WBC: White blood cell, *109/L. HB: hemoglobin, g/L. Neutrophil: *109/L. CRP: C-reactive protein. BNP: Brain natriuretic peptide. cTnI: Cardiac troponin I, ng/mL. RBC: Red blood cell, *1012/L. TC: Total cholesterol, mmol/L. TG: Triglyceride, mmol/L. LDL: Low density lipoprotein, mmol/L. HDL: High density lipoprotein, mmol/L. Scr: Serum creatinine. BUN: Blood urea nitrogen. eGFR: estimate glomerular filtration rate, mL/min/1.73m^2^. HP: Hypertension. DM: Diabetes mellitus. LM: Left Main Artery. LAD: Left anterior descending branch. LCX: Left Circumflex Artery. RCA: Right coronary artery. PCI: Percutaneous coronary intervention. ACEI/ARB: Angiotensin converting enzyme inhibitors/ Angiotensin receptor blocker. LMWH: Low molecular weight heparins.

### 3.2 Long term clinical outcomes

**Figure 3:**
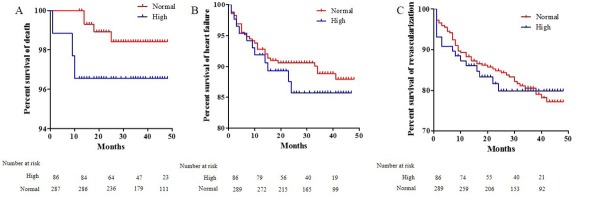
Kaplan-Meier survival analysis of MACEs. A, cumulative hazard ratio of death between two groups. B, cumulative hazard ratio of heart failure between two groups. C, cumulative hazard ratio of revasclarization between two groups.

Four patients died during 12-48 (average 25 months) months follow-up in normal neutrophil group compared with 3 patients died in high neutrophil group (hazard ration [HR] = 2.678; 95% confidence interval [95% CI] = 1.599-11.969; P=0.019). Fifty-five patients in normal neutrophil group and 16 patients in high neutrophil group underwent revascularization, respectively (HR = 1.047; 95% CI = 0.600-1.827; P=0.872). Heart failure occurred in 31 patients in normal neutrophil group and 11 in high neutrophil group, respectively (HR = 2.268; 95% CI = 1.637-4.523; P=0.041). After adjusting for CRP, the HR for death was 2.174, 95% CI was 1.024-10.248, P=0.025. For revascularization, the adjusted HR was 2.357, 95% CI was 0.578-4.214, P=0.357. For heart failure, the adjusted HR was 2.689, 95% CI was 1.217-6.248, P=0.037 (**Table 2**). The mean level of BNP at end of follow-up in normal neutrophil group and high neutrophil group was 218±32 pg/mL, 317±41 pg/mL, respectively. The mean ejection fraction at end of follow-up in normal neutrophil group and high neutrophil group was 49±7%, 41±6%, respectively (**Figure 2**). Kaplan-meier survival analysis showed that there are no difference in the cumulative hazard of deaths from all causes (P=0.018), revascularization (P=0.497), and heart failure (P=0.037) between two groups (**Figure 3**).

### 3.3 Subgroups analysis

Further, a subgroups analysis was performed according to renal function stratification. Three subgroups were specified as eGFR 65-90, 40-64 and 15-39 mL/min/1.73m^2^, respectively. The primary outcome of revascularization was consistent across all subgroups (**Table 3**). The data showed that high neutrophil count has no effect s on increasing the ratio of revascularization no matter the grade of renal dysfunction.

**Table 3:** Hazard ratios for the revascularization in subgroups of patients.

subgroup	Normal no of events/ total patients	High no of events/ total patients	Hazard ratio(95%CI)	*P*	Adjusted Hazard ratio^a^ (95% CI)	*P*
**eGFR (mL/min/1.73m^2^)**						
≥65	41/209	11/55	0.899(0.462-1.749)	0.753	0.978(0.258-2.367)	0.325
40-64	13/61	3/21	0.739(0.210-2.596)	0.637	0.874(0.124-3.687)	0.429
15-39	1/20	2/11	3.876(0.351-42.771)	0.269	1.238(0.487-11.368)	0.147

HR was calculated as normal group was control group. CI: Confidence interval. no: Number. a: adjusted for CRP.

## 4 Discussion

In this retrospective cohort study from a real-world clinical practice, a high proportion of neutrophil leads to higher incidence in death from all causes and heart failure. However, the proportion of neutrophil failed to indicate the long term revascularization in STEMI patients with chronic renal dysfunction. Neutrophil count measurement is simple and low cost and is widely used in clinical practice. This is the first time that correlation between neutrophil count and prognosis of STEMI patients with chronic renal dysfunction has been shown. The data showed that neutrophil count is a potential marker to indicate the prognosis of STEMI in chronic kidney disease.

A great number of studies showed that inflammation participate in pathological progress of atherosclerosis. In the setting of MI, chemokines could recruit WBC to the sites of ischemia [10]. The accumulation of high concentration of chemokines at the ischemic site lead to the injury of infarction area. Different subtypes of WBC may play different role in inflammatory reaction after myocardial infarction [11, 12]. Neutrophils produce several inflammatory mediators that cause acute myocardial injury or further tissue damage after STEMI. The bigger amount of neutrophils always lead to larger area of infarction and worse prognosis [13, 14, 15]. Moreover, the quantity of neutrophils may lead to microcirculatory disturbance. Previous research shows that neutrophil is an independent risk factor of short-term death and long-term death in myocardial infarction patients [16, 17].

**Table 2:** Hazard ratio of MACEs according to two groups.

	Patients (n=377)			P		
	Normal (n=290)	High (n=87)	HR (95% CI)		Adjusted HR (95% CI)^a^	P
Death						
no./total no.	4/290	3/87	2.678	0.019	2.174	0.025
(%)	(1.4)	(3.4)	(1.599-11.969)		(1.024-10.248)	
Revascularization			1.047	0.872	2.357	0.357
no./total no.	55/290	16/87	(0.600-1.827)		(0.578-4.214)	
(%)	(19.0)	(18.4)	2.268	0.041	2.698	0.037
Heart failure			(1.637-4.523)		(1.217-6.248)	
no./total no.	31/290	11/87				
(%)	(10.7)	(12.6)				

Normal: normal neutrophil count group. High: high neutrophil count group. a: adjusted for CRP.

A great number of studies showed that neutrophil may predict long term prognosis of myocardial infarction. YC Han *et al*. [2] found that neutrophil to lymphocyte ratio is a useful marker to predict 12-month MACE and death in patients with STEMI who have undergone primary PCI. Moreover, Hc Shin *et al*. [7] suggested that elevated levels of both NLR and CRP are associated with increased risk of long-term mortality in myocardial infarction patients who have undergone PCI. Additionally, Karetnikova *et al*. reported that serum neutrophil gelatinase-associated lipocalin was associated with the existing adverse outcomes and can serve as biomarker of MI severity [18]. This research indicated that neutrophil is an independent factor for predicting clinical outcomes of myocardial infarction. Our study focuses on the specific myocardial infarction population who suffered from chronic renal dysfunction. The results also show that neutrophil increases the incidence of death, heart failure and revascularization. However, there were no statistical differences in revascularization.

In chronic renal dysfunction patients, hypercoagulable state result in increasing incidence of myocardial infarction and other thrombosis [19]. High oxidative stress are associated with mitochondrial dysfunction and then lead to injury of endothelial cells [20, 21, 22]. Inflammation is also a critical factor involved in renal dysfunction. High neutrophil count is common in patients with renal dysfunction. Previous researches indicate that renal dysfunction is related with high incidence of death and heart failure. Interestingly, our study showed that neutrophil exhibit positive role in predicting the prognosis of myocardial infarction with renal dysfunction, except for long term revascularization. It has been proved that renal dysfunction is associated with poor prognosis of myocardial infarction, for example, isocheimal events [23, 24]. Intensely inflammatory reaction counteracting the predicting value of neutrophil. Moreover, a great number of patients with chronic renal dysfunction have not received the standard therapy when myocardial infarction happening [25], which may be another possible cause.

Above all, chronic renal dysfunction showed a close relationship with poor prognosis in myocardial infarction. Neutrophil is a predictive factor that predicting long term outcomes of myocardial infarction. In chronic dysfunction patients, the high level neutrophil has statistical relation with increased incidence of death and heart failure. However, it failed to predict the incidence of revascularization in 12-48 months.

There are some obvious limitations in this study. First, the detailed drug therapy during follow-up was not collected. Second, it is a retrospective study that the sample sized is not accurately calculated.

## 5 Conclusion

Neutrophil counts predict the clinical outcomes in myocardial infarction patients with chronic renal dysfunction.
